# Ursodeoxycholic Acid (UDCA) Exerts Anti-Atherogenic Effects by Inhibiting RAGE Signaling in Diabetic Atherosclerosis

**DOI:** 10.1371/journal.pone.0147839

**Published:** 2016-01-25

**Authors:** Jihwa Chung, Shung Hyun An, Sang Won Kang, Kihwan Kwon

**Affiliations:** 1 Medical Research Institute, School of Medicine, Ewha Womans University, Seoul, Korea; 2 Department of Internal Medicine, Cardiology Division and GT5 Program of Ewha Womans University School of Medicine, Seoul, Korea; 3 Center for Cell Signaling Research and Division of Molecular Life Sciences, Ewha Womans University, Seoul, Korea; University of Miami, UNITED STATES

## Abstract

A naturally occurring bile acid, ursodeoxycholic acid (UDCA), is known to alleviate endoplasmic reticulum (ER) stress at the cellular level. However, the detailed action mechanisms of UDCA in atherosclerosis are not fully understood. In this study, we demonstrated whether UDCA exerts anti-atherogenic activity in diabetic atherosclerosis by targeting ER stress and “receptor for advanced glycation endproduct” (RAGE) signaling. UDCA markedly reduced ER stress, RAGE expression, and pro-inflammatory responses [including NF-κB activation and reactive oxygen species (ROS) production] induced in endothelial cells (ECs) by high glucose (HG). In particular, UDCA inhibited HG-induced ROS production by increasing the Nrf2 level. In macrophages, UDCA also blocked HG-induced RAGE and pro-inflammatory cytokine expression and inhibited foam cell formation via upregulation of the ATP-binding cassette (ABC) transporters, ABCA1 and ABCG1. In the diabetic mouse model, UDCA inhibited atheromatous plaque formation by decreasing ER stress, and the levels of RAGE and adhesion molecules. In conclusion, UDCA exerts an anti-atherogenic activity in diabetic atherosclerosis by targeting both ER stress and RAGE signaling. Our work implicates UDCA as a potential therapeutic agent for prevention or treatment of diabetic atherosclerosis.

## Introduction

Diabetes is associated with accelerated atherosclerosis in humans; this is a major cause of morbidity and mortality [1, 2]. An important mechanism by which hyperglycemia contributes to accelerated atherosclerosis is via extensive formation of advanced glycation end products (AGEs) [3], which are products of nonenzymatic glycation and oxidation of proteins and lipids. The transmembrane receptor for advanced glycation end products (RAGE) recognizes AGEs and other ligands including S100/calgranulin and HMGB1 (high-mobility group box 1) protein [4]. Interaction of RAGE with AGEs plays a pivotal role in regulating the production/expression of inflammatory cytokines, oxidative stress, and endothelial dysfunction, in diabetes [5]. The principal mechanisms involved in many cell types are induction of ER stress [6] and activation of nuclear factor κB (NF-κB) [4, 7].

Many studies have found that activation of the unfolded protein response (UPR) in the ER, referred to as ER stress, plays fundamental roles in the development and progression of atherosclerosis[8–10]. ER stress is an adaptive response that seeks to maintain ER homeostasis but, if the stress remains unresolved, apoptotic cell death and ROS generation may follow. The UPR signaling cascade is initialized by activation of three ER-resident proteins: activating transcription factor-6 (ATF6), inositol requiring protein-1 (IRE1), and protein kinase RNA-like ER kinase (PERK) [11, 12]. Also, the UPR and inflammation are interconnected via various mechanisms, including ROS production, release of calcium from the ER, and activation of NF-κB and the mitogen-activated protein kinase (MAPK) known as JNK (Jun N-terminal kinase) [12].

Massive oxLDL uptake by macrophages triggers foam cell formation, a critical step in development of atherosclerosis, caused by an imbalance between cholesterol influx and efflux. The scavenger receptor CD36 and the ATP binding cassette (ABC) transporter family, ABCA1 and ABCG1, are known regulators of cholesterol influx and efflux. Much evidence indicates that the level of the scavenger receptor CD36 increases and those of ABC transporters (such as ABCA1 and ABCG1) decrease significantly in patients with diabetes and vascular complications thereof, such as atherosclerosis [13, 14].

UDCA is a hydrophilic tertiary bile salt that is widely used to treat chronic cholestatic liver disease and is beneficial when given to patients with various liver diseases, including primary biliary cirrhosis and chronic viral hepatitis [15, 16]. UDCA exhibits a wide range of cellular actions, including anti-apoptotic and anti-inflammatory effects, but these have previously been described only in hepatocytes. Interestingly, a previous study found that UDCA increased nitric oxide production and inhibited endothelin-1 production in human vascular ECs [17], suggesting that UDCA might exert cytoprotective effects on such cells. In animals, chemical chaperones, including 4-phenylbutyric acid and taurin-conjugated ursodeoxycholic acid (TUDCA), have been shown to alleviate ER stress, and to act as potent anti-diabetic agents in diabetic mice [18]. Also, TUDCA inhibits neointimal hyperplasia by reducing the proliferation of and inducing apoptosis in vascular smooth muscle cells of rats subjected to carotid artery balloon injury [19]. However, the effects and mechanisms of action of UDCA in the context of diabetic vascular complications such as atherosclerosis are not fully understood.

In the present study, we show that UDCA exerts anti-atherogenic effects on both ECs and macrophages under hyperglycemic conditions. UDCA inhibited the development of atherosclerotic lesions (via suppression of endothelial dysfunction), and foam cell formation by macrophages. This was because UDCA inhibited RAGE signaling. Therefore, UDCA may be a valuable therapeutic agent for prevention or treatment of diabetic atherosclerosis.

## Materials and Methods

### Reagents and materials

UDCA was kindly provided by Daewoong Pharmaceutical Co. Ltd. (Seoul, Korea). D-glucose, mannitol, streptozotocin (STZ), pyrrolidine dithiocarbamate (PDTC), and an NF-κB inhibitor were purchased from Sigma-Aldrich (St Louis, MO). SP600125, a selective JNK inhibitor, was from Promega (Madison, WI). Antibodies against phospho-IκB, NF-κB p65, phospho-ERK_1/2_, ERK_1/2_, phospho-JNK, and JNK were from Cell Signaling Technology (Beverly, MA). Antibodies against RAGE, S100A12, and XBP1 were from Abcam (Cambridge, MA). Anti-phospho-PERK, anti-CHOP, anti-Nrf2, anti-phospho-p38, anti-p38, anti-β-actin, anti-CD68, anti-CD11b/c, anti-GRP78, anti-mouse IgG-R, and anti-rabbit IgG-R antibodies were from Santa Cruz Biotechnology (Santa Cruz, CA). Anti-ATF6 antibody was from IMGENEX (San Diego, CA). Anti-VCAM-1 antibody was from Novus Biologicals. Anti-ICAM-1 antibody was from SouthernBiotech (Birmingham, AL). Anti-rat IgG-R antibody was from Jackson ImmunoResearch (West Grove, PA).

### Cell culture, exposure to high glucose (HG), and UDCA treatment

Primary HUVECs (Human Umbilical Vein Endothelial cells) were isolated from umbilical cord of a newborn as described previously [20], and cells in passages 3–5 were used in this study. Cells were cultured in Medium 200 with 5% (v/v) fetal bovine serum and low-serum growth supplement (LSGS; Life technologies., Grand Island, NY) [21]. Cells of the human leukemic monocyte lymphoma cell line U937 were cultured in RPMI-1640 medium with 10% (v/v) FBS at 37°C under 5% (v/v) CO_2_. Cells of the mouse macrophage cell line Raw264.7 were cultured in DMEM (HyClone Laboratories Inc., South Logan, Utah) with 10% (v/v) FBS. D-glucose was dissolved in water, and 30 mM was considered to reflect high glucose (HG) conditions [22]. Mannitol (M, 30 mM) was used as osmotic control. UDCA was dissolved in DMSO and administered for the indicated times at various concentrations.

### Western blotting

Cells were harvested in lysis buffer containing 0.1% (v/v) of a protease inhibitor mixture (Roche Diagnostics, Mannheim, Germany). Protein concentrations in cell lysates were measured with the aid of a BCA protein assay kit (Thermo Scientific, Rockford, IL). Equal amounts of protein were subjected to SDS-PAGE gel electrophoresis and transferred to nitrocellulose membranes; Western blotting was next performed as previously described [23]. After protein detection, membranes were stripped of bound antibody and reprobed with an anti-β-actin antibody.

### Preparation of cytoplasmic and nuclear extracts

Nuclear and cytoplasmic extracts were prepared with the aid of a Nuclear/Cytosol Fractionation Kit (BioVision Inc., Mountain View, CA) following the manufacturer's instructions. All steps were performed on ice. Briefly, cell pellets were suspended in CEB-A buffer containing DTT and protease inhibitors. After 10 min, CEB-B buffer was added and incubation continued for 1 min. Nuclei were collected by centrifugation at 16,000 g for 5 min. The supernatants (cytosol extracts) were also collected. The pellets were resuspended in NEB buffer and incubated for 40 min. Nuclear proteins were obtained by centrifugation at 16,000 g for 10 min and the supernatants (nuclear extracts) retained. All extracts were stored at −80°C prior to analysis.

### Reverse transcription chain reaction (RT-PCR) and real time quantitative RT-PCR (qRT-PCR)

Total cellular RNA was extracted from cultured HUVECs and Raw264.7 cells using a Total RNA Isolation Kit (Qiagen, Inc., Valencia, CA) according to the manufacturer’s instructions [21]. First-strand complementary DNA (cDNA) was synthesized with the aid of M-MLV reverse transcriptase (Promega, Madison, WI) and oligo-dT 15 primer (Promega, Madison, WI). cDNA was amplified with Mastercycler (Eppendorf, Hamburg, Germany). Real-time qRT-PCR was performed with the aid of SYBR Green PCR Master Mix (Qiagen, Inc., Valencia, CA) on an ABI StepOne Real-time PCR System (Applied Biosystems, CA). The primers are shown in S1 Table.

### Monocyte adhesion assay

HUVECs were pretreated with UDCA (100 μM) for 2 h followed by exposure to HG for 24 h. As a positive control, tumor necrosis factor-α (TNF-α, 10 ng/mL) was added for 6 h. U937 cells were added to HUVECs followed by incubation for 30 min at 37°C. Unbound cells were next removed by washing in serum-free medium [24]. Adherent cells were counted in five randomly selected optical fields of each well. Phase-contrast microphotographs of cells in plates were taken with an Olympus CKX41 microscope (Olympus America, Melville, NY).

### Foam cell formation by macrophages

Oil-red-O staining was used to detect foam cell formation. Raw264.7 cells were incubated with 50 μg/mL oxLDL (KALEN Biomedical, Inc., Savage, MD), with or without UDCA for 24 h. Cells were fixed in 4% (v/v) paraformaldehyde and stained with Oil-red-O to quantify foam cell numbers.

### Detection of intracellular ROS

ROS generation in cells was measured with the aid of CM-H_2_DCF-DA (Molecular Probes, Eugene, OR). Cells were pretreated with UDCA for 2 h, exposed to HG for 48 h, washed in phenol red-free Medium 200, and incubated with freshly prepared 5 μM CM-H_2_DCF-DA for 5 min. All samples were separately stained for the same amount of time. The cells were next washed twice in phenol red-free Medium 200 and examined via fluorescence microscopy [25].

### The in vivo mouse model

This study was carried out in accordance with IACUC guidelines and approved by the Committee on Ethics in Animal Experiments at Ewha Womans University (Permit number: 12–0203). All animal works was performed under anesthesia with zoletil-rompun mixture, and all efforts were made to minimize suffering. As a model of diabetic atherosclerosis, diabetes was induced in male ApoE^-/-^ mice (7–9 weeks of age) via injections of 50 mg/kg STZ (50 mM sodium citrate, pH 4.5) on each of 5 successive days, after 8 h of fasting. Animal with blood glucose levels > 250 mg/dL were considered diabetic, and were divided into two groups: a control group fed a chow diet and a UDCA-treated group fed a chow diet with 0.5% (w/v) UDCA, both for 10 weeks. Aortas were then collected for Oil-red-O staining and immunohistochemistry.

### Immunofluorescence staining

Aortic tissues were embedded in OCT medium and frozen. Sections 7 μM in thickness were prepared. After fixation in 4% (v/v) paraformaldehyde, the sections were blocked with 10% (v/v) normal goat or donkey serum, in PBS, for 1 h. Tissue sections were stained with rabbit anti-phospho-PERK (1:50), rabbit anti-XBP-1 (1:50), mouse anti-ATF6 (1:50), rabbit anti-RAGE (1:50), rabbit anti-VCAM1 (1:50), rat anti-ICAM1 (1:50), rabbit anti-CD68 (1:50), or rabbit anti-CD11b/c (1:50), overnight at 4°C. Samples were incubated for 2 h with appropriate secondary antibodies (anti-mouse IgG-R, anti-rabbit IgG-R, or anti-rat IgG-R) in the dark. DAPI (100 ng/mL; Santa Cruz Biotechnology) was used for nuclear counterstaining. After mounting, tissue slides were observed with the aid of an Olympus BX51 microscope (Olympus America, Melville, NY).

### Quantitation of atherosclerotic lesions

Briefly, aortas from diabetic ApoE^-/-^ mice fed a chow diet with or without 0.5% (w/v) UDCA were harvested after perfusion with saline and then immediately fixed in 4% (v/v) formaldehyde. Whole aortas were opened lengthwise and stained with Oil-red-O [26]. The sizes of atherosclerotic lesions were measured using the Image J software.

### Statistical analysis

All data are expressed as means ± SEMs and represent data from at least three independent experiments. The overall comparison of relative expression levels among four groups was performed with Kruskal-Wallis test. If the overall comparison was significant at P < 0.05, the post-hoc pairwise comparisons between two groups (control vs. HG/TG and HG/TG vs. HG/TG + UDCA) were performed by Mann-Whitney U test with Bonferroni adjustment. A P value < 0.025 was considered statistically significant in the post-hoc Mann-Whitney U test. In STZ-induced diabetic mice, the comparison between STZ and STZ +UDCA group was performed with Mann-Whitney U test. Differences between STZ and STZ +UDCA group were considered to be significant at P < 0.05.

## Results

### UDCA suppresses expression of RAGE and the ligands thereof by inhibiting HG-induced ER stress in ECs

**Fig 1:**
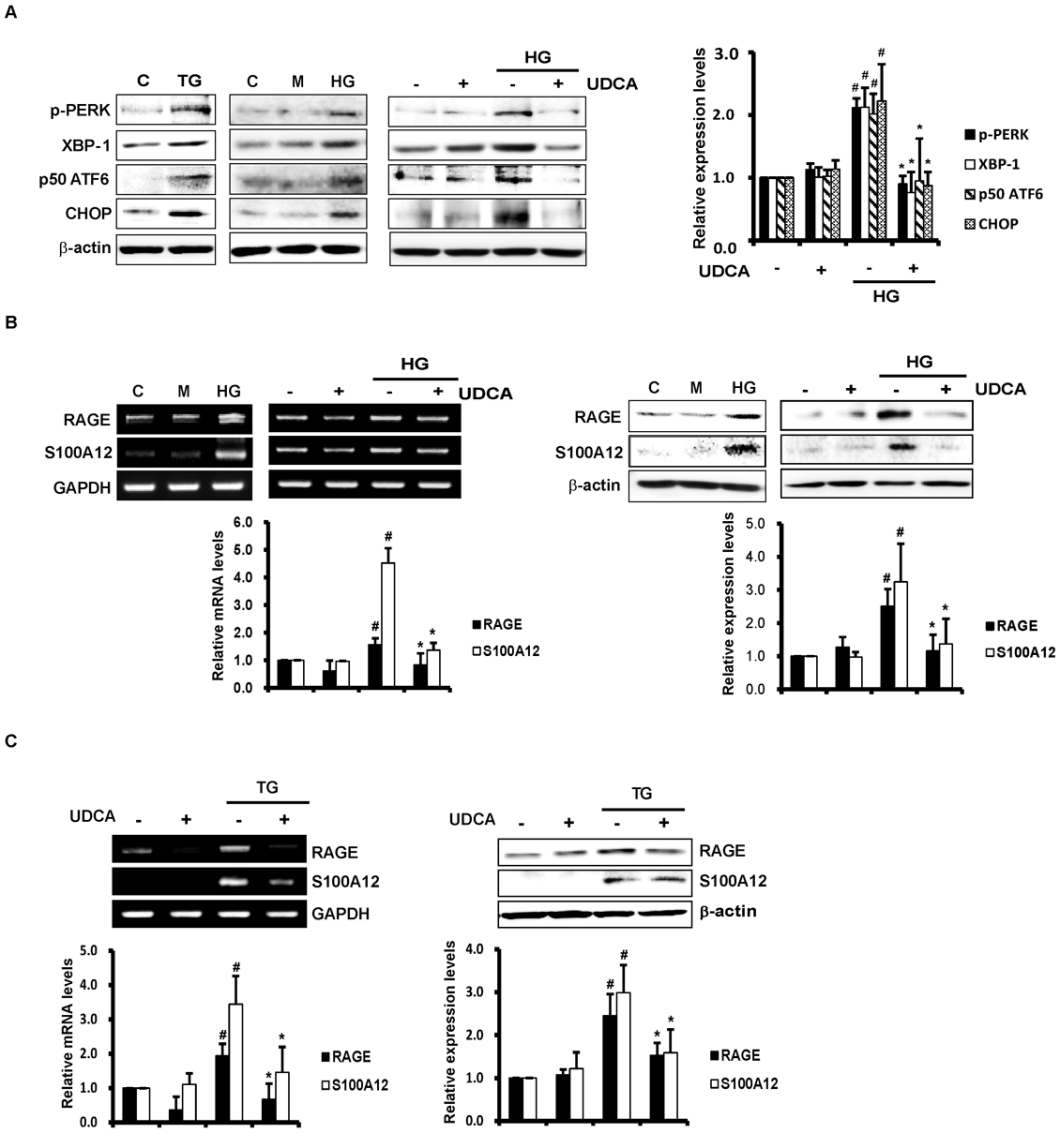
Effects of UDCA on ER stress and expression of RAGE and the ligand thereof. Cells were incubated with HG (30 mM) or mannitol (M; 30 mM, osmotic control) for varying periods after pretreatment with UDCA (100 μM) for 2 h. (**A**) The expression levels of ER stress markers including phospho-PERK, XBP-1, p50 ATF6, and CHOP were measured by Western blotting. Thapsigargin (TG, 2 μM) was used as a positive control (triggering ER stress). #,* Significant difference at the P < 0.025 based on post-hoc Mann-Whitney U test following a Kruskal-Wallis test (#, Control vs. HG; *, HG vs. HG + UDCA). Error bars: SEMs. (**B** and **C**) The levels of mRNAs encoding RAGE and the ligand thereof, and the protein levels per se, were detected by RT-PCR and Western blotting, respectively. RNA was extracted from cells after 8 h of HG or TG treatment. RT-PCR using primers amplifying *RAGE*, *S100A12*, and *GAPDH* (internal control) was performed as described in Methods and Materials. Total proteins were prepared from cell lysates after 24 h of HG (**B**) or TG (**C**) exposure. Representative images from at least three experiments are shown. #,* Significant difference at the P < 0.025 based on post-hoc Mann-Whitney U test following a Kruskal-Wallis test (#, Control vs. HG/TG; *, HG/TG vs. HG/TG + UDCA). Error bars: SEMs.

Many previous studies have shown that UDCA exerts anti-inflammatory and anti-apoptotic effects in hepatocytes via inhibition of ER stress. ER stress levels were measured by quantifying the levels of classical markers of the UPR pathway, including phospho-PERK, XBP-1, p50 ATF6, and CHOP. In the present study, we found that UDCA suppressed the ER stress induced by thapsigargin (TG), even in ECs (S1 Fig). As shown in Fig 1A, Western blotting showed that UDCA inhibited increases of HG-induced ER stress markers including phospho-PERK, XBP-1, p50 ATF6, and CHOP. In addition, both RT-PCR and Western blotting showed that the expression levels of RAGE and the S100A12 ligand thereof increased in ECs exposed to HG. In contrast, UDCA treatment significantly reduced HG-induced expression of RAGE and S100A12 (Fig 1B). In addition, Mannitol, used as an osmotic control, did not affect the expression of ER stress markers, RAGE and S100A12 ligand (Fig 1A and 1B). We explored direct crosstalk between RAGE and ER stress induced using TG. Interestingly, ER stress induced by TG was associated with markedly increased expression of the mRNAs encoding RAGE and S100A12. These results imply that ER stress directly induces RAGE expression in ECs. Furthermore, UDCA-treated ECs exhibited reduced expression of RAGE and S100A12 upon induction by TG, as well as under HG conditions (Fig 1C). These results suggest that, in ECs, UDCA can suppress RAGE expression by inhibiting ER stress induced by HG and/or direct ER stress inducers.

### UDCA suppresses HG-induced vascular inflammation in ECs

**Fig 2:**
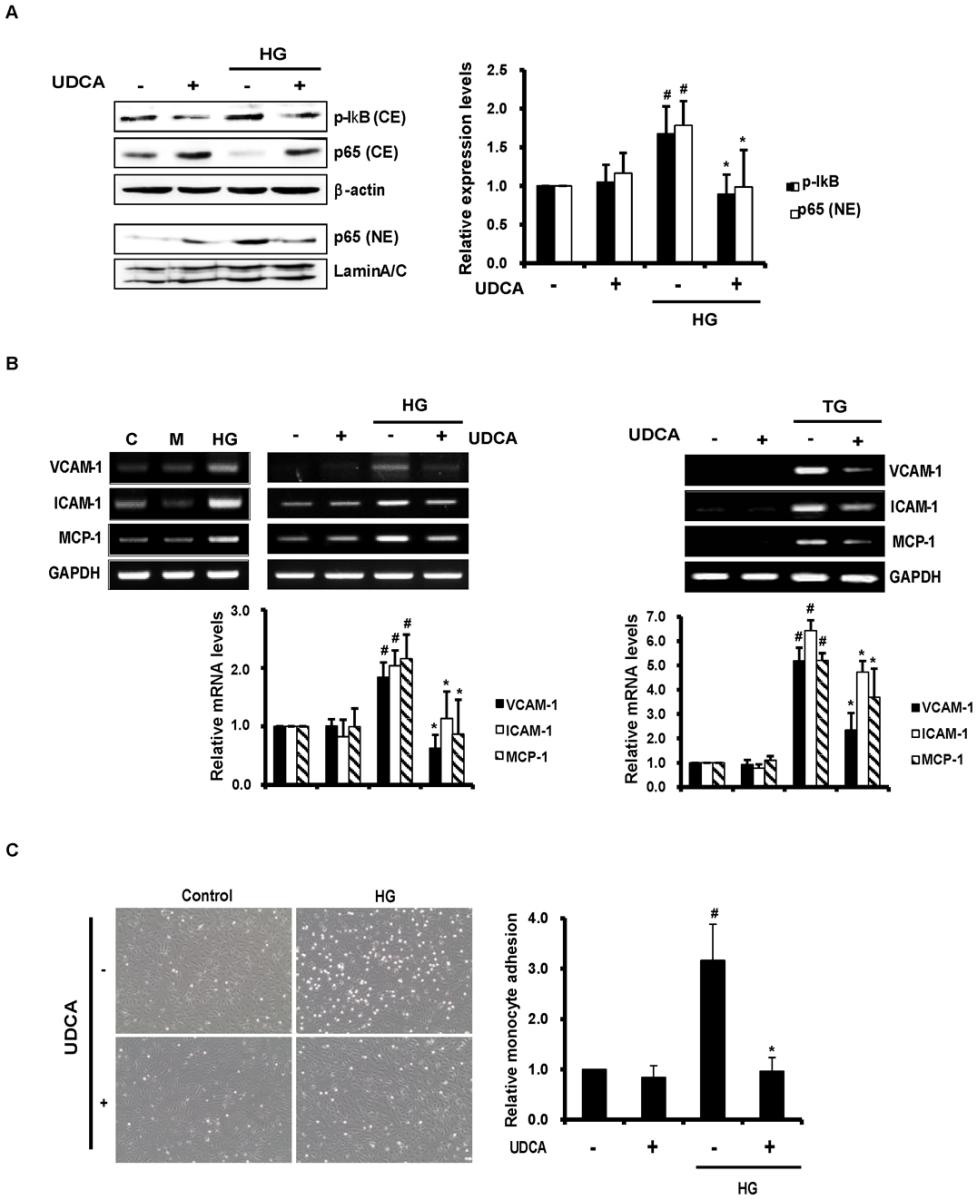
Effects of UDCA on NF-κB activation and the vascular inflammatory response. (**A**) Cytoplasmic and nuclear fractions were prepared after 1 h of HG. The extents of IκB phosphorylation and nuclear translocation of NF-κB were determined by Western blotting. Representative images from at least three experiments are shown. CE, cytoplasmic extracts; NE, nuclear extracts. #,* Significant difference at the P < 0.025 based on post-hoc Mann-Whitney U test following a Kruskal-Wallis test (#, Control vs. HG; *, HG vs. HG + UDCA). Error bars: SEMs. (**B**) RNAs were extracted from cells after 8 h of HG, M (osmotic control) or TG exposure. The levels of mRNAs encoding VCAM-1, ICAM-1, and MCP-1 were determined by RT-PCR. Representative images from at least three experiments are shown. #,* Significant difference at the P < 0.025 based on post-hoc Mann-Whitney U test following a Kruskal-Wallis test (#, Control vs. HG/TG; *, HG/TG vs. HG/TG + UDCA). Error bars: SEMs. (**C**) Monocyte (U937) adhesion assay. Cells were exposed to HG for 24 h after pretreatment with UDCA. U937 cells were added to ECs and adherent monocytes counted in five random optical fields in each dish (magnification, X 100; scale bars, 100 μm). #,* Significant difference at the P < 0.025 based on post-hoc Mann-Whitney U test following a Kruskal-Wallis test (#, Control vs. HG; *, HG vs. HG + UDCA). Error bars: SEMs.

Activation of NF-κB is mediated via phosphorylation of the inhibitory subunit thereof, IκB. In the present study, I*κ*B phosphorylation increased in HG-treated ECs, and this was inhibited by UDCA. Nuclear translocation of p65 NF-*κ*B increased in HG-treated ECs, and UDCA also inhibited this process (Fig 2A). In addition, we explored the effects of UDCA on downstream signaling by NF-*κ*B; we measured the expression levels of various adhesion molecules (VCAM-1, ICAM-1, and MCP-1). As expected, the increases in the levels of mRNAs encoding adhesion molecules, triggered by HG condition, were significantly inhibited by UDCA. Mannitol-treated ECs showed no changes in expression levels of adhesion molecules. UDCA-treated ECs also exhibited significant reductions in the levels of adhesion molecules associated with ER stress induced by TG (Fig 2B). As shown in Fig 2C, adhesion of monocytes to ECs was markedly increased by HG, but was significantly inhibited by UDCA. These results show that UDCA exerts anti-inflammatory effects via suppression of NF-*κ*B activation and inhibition of the expression of adhesion molecules induced by HG and ER stress.

### UDCA attenuates HG-induced oxidative stress

**Fig 3:**
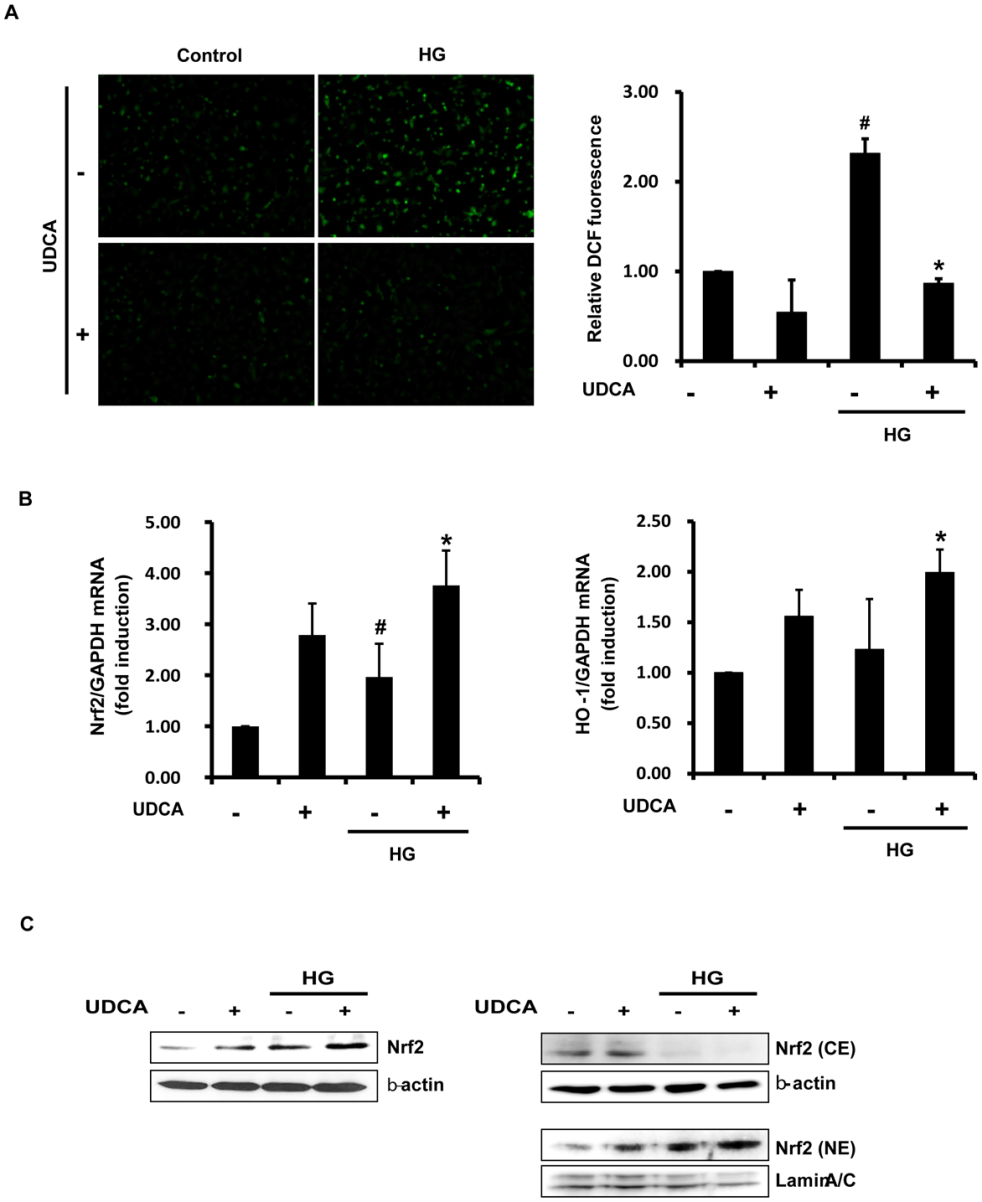
Effects of UDCA on HG-induced oxidative stress. (**A**) ROS production in cells after 48 h of HG detected by DCF staining and fluorescent microscopy (magnification, X 100; scale bars, 100 μm). The intensity of fluorescence was quantified using the Image J software. #,* Significant difference at the P < 0.025 based on post-hoc Mann-Whitney U test following a Kruskal-Wallis test (#, Control vs. HG; *, HG vs. HG + UDCA). Error bars: SEMs. (**B**) The levels of mRNAs encoding antioxidant proteins, Nrf2 and HO-1, were measured after 2 h of HG, by real time-qPCR. #,* Significant difference at the P < 0.025 based on post-hoc Mann-Whitney U test following a Kruskal-Wallis test (#, Control vs. HG; *, HG vs. HG + UDCA). Error bars: SEMs. (**C**) Nuclear translocation of Nrf2 was detected by Western blotting. The cytoplasmic (CE) and nuclear fractions (NE) were prepared after 1 h of HG. Representative images from at least three experiments are shown.

ROS generation in ECs under HG conditions was measured via DCF fluorescence staining. ROS production was enhanced in ECs under such conditions, and was suppressed by UDCA (Fig 3A). To determine the mechanism involved in ROS reduction in UDCA-treated ECs, we used RT-PCR to measure the expression levels of mRNAs encoding anti-oxidant proteins [including haem oxygenase-1 (HO-1) and superoxide dismutase-1 (SOD-1)]; enzymes involved in glutathione biosynthesis [including γ-glutamylcysteine ligase catalytic (GCLC), modulatory (GCLM) subunits, and glutathione synthase (GSHS)]; and transcription factors [including nuclear factor erythroid-2-related factor-2 (Nrf2)]. The levels of mRNAs encoding HO-1 and Nrf2 increased under HG (Fig 3B), but the levels of those encoding SOD-1, GCLC, GCLM, and GSHS were not affected (S2 Fig). HG increased the expression level of Nrf2 mRNA to compensate for oxidative stress, and UDCA triggered a much greater increase in Nrf2 mRNA than did HG. The expression level of HO-1 was correlated with the Nrf2 expression level under both HG and in UDCA-treated ECs. In addition, Western blotting showed that UDCA increased the expression level of Nrf2 per se in whole-cell lysates and, more interestingly, promoted nuclear translocation of Nrf2 to a greater extent than noted under HG in the absence of UDCA (Fig 3C). These results suggest that UDCA attenuates HG-induced oxidative stress by regulating Nrf2 expression.

### UDCA suppresses HG-induced RAGE expression and RAGE signaling

**Fig 4:**
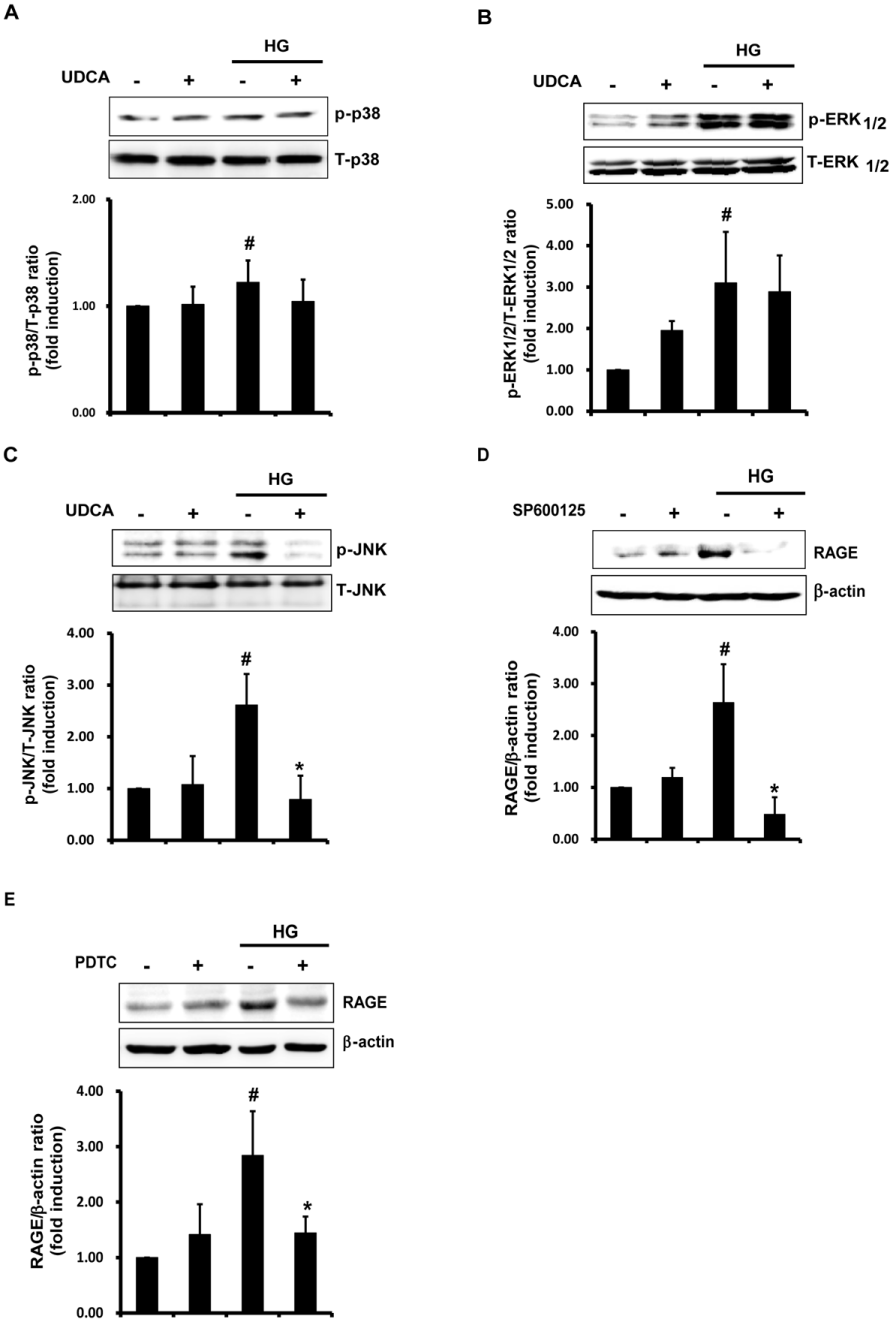
Effects of UDCA on activation of the p38 MAPK, ERK1/2, and JNK signaling pathways induced by HG. The phosphorylated and total levels of p38 MAPK (**A**), ERK_1/2_ (**B**), and JNK (**C**) in cells under HG (for 24 h) were measured by Western blotting. The ratios of phosphorylated to total protein levels were determined using the Image J software. #,* Significant difference at the P < 0.025 based on post-hoc Mann-Whitney U test following a Kruskal-Wallis test (#, Control vs. HG; *, HG vs. HG + UDCA). Error bars: SEMs. (**D** and **E**) The JNK inhibitor SP600125 (10 μM) or the NF-κB inhibitor PDTC (10 μM) were added for 1 h prior to HG. Western blotting was used to detect RAGE. Representative images from at least three experiments are shown. #,* Significant difference at the P < 0.025 based on post-hoc Mann-Whitney U test following a Kruskal-Wallis test (#, Control vs. HG; *, HG vs. HG + SP600125/ PDTC). Error bars: SEMs.

Activation of ERK (extracellular signal-regulated kinase)_1/2_, p38 MAPK, and JNK, triggers NF-κB activation [6]. ER stress also can induce NF-κB activation via IRE1α-JNK activation. As UDCA exerted inhibitory effects on HG-induced NF-κB activation in the present study, we sought to identify the pathway responsible for suppression of NF-κB activation by UDCA. The activation levels of p38 MAPK, ERK_1/2_, and JNK were calculated as the ratios between the levels of phosphorylated and total p38, ERK_1/2_, and JNK kinases, respectively, as revealed by Western blotting. HG increased phosphorylation of p38 MAPK, ERK_1/2_ and JNK (Fig 4A–4C). UDCA exerted an inhibitory effect on JNK activation induced by HG, but not on p38 MAPK and ERK_1/2_ activation. Moreover, UDCA per se induced phosphorylation of ERK_1/2_ to an extent greater than the control (Fig 4B). Some reports have indicated that ERK_1/2_ acts within a signaling cascade that regulates various cellular processes including proliferation, differentiation, and cell cycle progression, in response to a variety of extracellular signals [27]. Consistent with this view, we confirmed that UDCA promoted endothelial proliferation and expression of mRNA encoding eNOS (S3 Fig). To explore the potential involvement of JNK pathways in terms of the reduction in HG-induced RAGE expression mediated by UDCA, we measured RAGE expression levels in the presence of an inhibitor of JNK, SP600125. Stimulation of RAGE expression in HG-treated ECs was inhibited by SP600125 (Fig 4D). Next, we explored whether RAGE expression induced by HG was attributable to NF-κB activation. The increase in RAGE expression induced by HG was diminished by PDTC, an inhibitor of NF-κB (Fig 4E). These results suggest that the UDCA-triggered reductions in HG-induced expression of RAGE and ligands thereof are mediated via inhibition of JNK and NF-κB activation.

### UDCA reduces foam cell formation and expression of pro-inflammatory cytokines in macrophages

**Fig 5:**
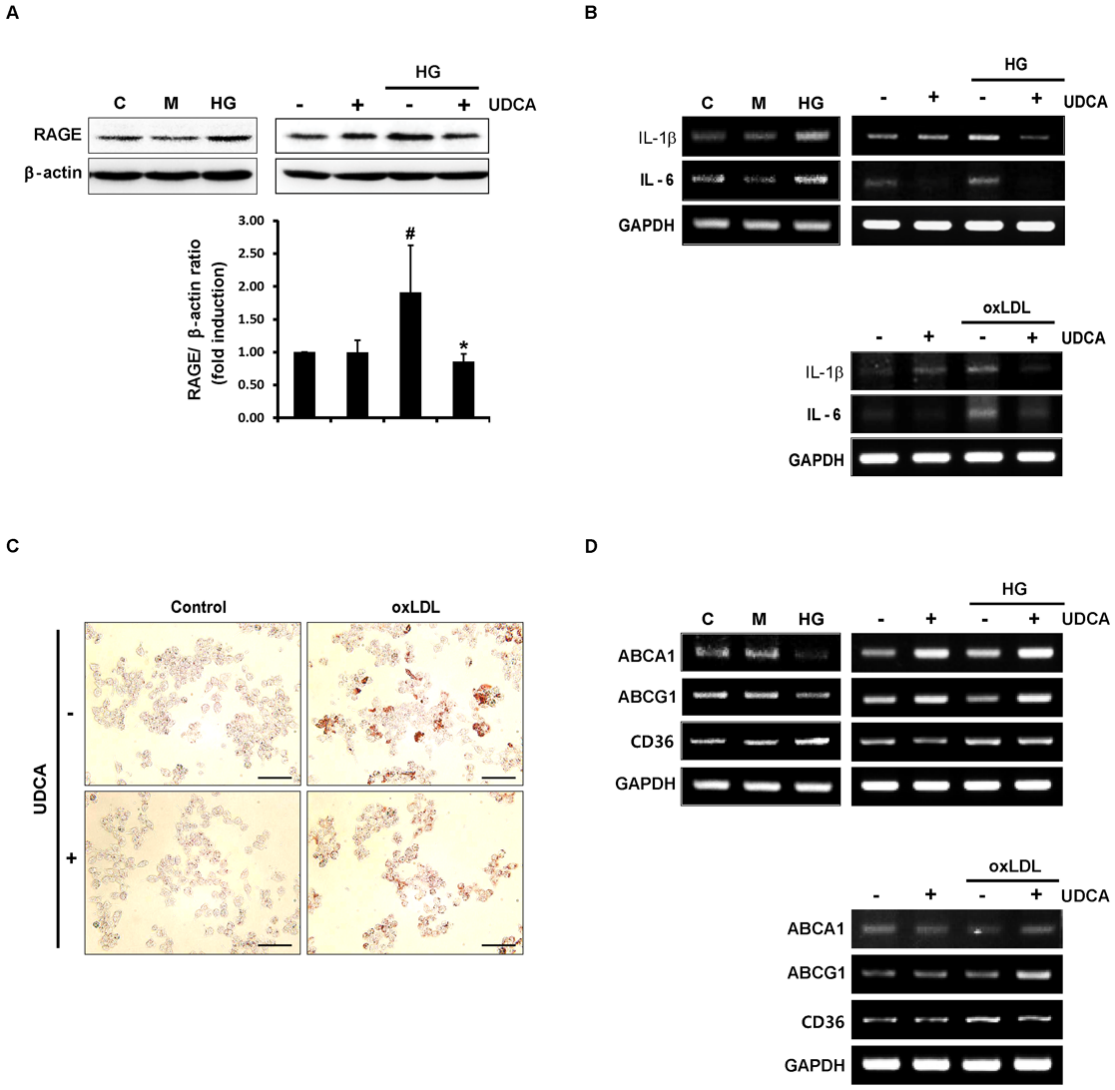
Effects of UDCA on foam cell formation by and inflammatory responses of macrophages. (**A**) Raw 264.7 cells were treated with UDCA for 2 h prior to HG or M (osmotic control) (for 24 h). RAGE levels were measured via Western blotting. #,* Significant difference at the P < 0.025 based on post-hoc Mann-Whitney U test following a Kruskal-Wallis test (#, Control vs. HG; *, HG vs. HG + UDCA). Error bars: SEMs. (**B** and **D**) RNAs were extracted after 6 h of HG, M or oxLDL. The levels of mRNAs encoding pro-inflammatory cytokines (IL-1β and IL-6) (B); the scavenger receptor CD36 and the ABC transporters (ABCA1 and ABCG1) (D), were measured via RT-PCR. Representative images from at least three experiments are shown. (**C**) Cells were treated with oxLDL (50 μg/mL) for 24 h after pretreatment with UDCA, and next fixed and stained with Oil Red O to quantify foam cell formation by macrophages. Representative images from at least three experiments are shown (magnification, ×100; scale bars, 100 μm).

Activation of RAGE signaling can trigger elevations in the levels of pro-inflammatory cytokines including IL-1β, IL-6, and TNF-α. In the present study, we showed that UDCA reduced the HG-induced expression of RAGE in macrophages (Fig 5A). As shown in Fig 5B, the HG- and oxLDL-induced elevations in the levels of mRNAs encoding IL-1β and IL-6, were remarkably reduced by UDCA. It was earlier shown that foam cell formation was reduced in RAGE knock-out mice [28]. As UDCA reduced the HG-induced expression of RAGE in macrophages in the present study, we next explored the effects of UDCA on foam cell formation. As a result, we found that UDCA reduced oxLDL-induced foam cell formation (Fig 5C). It has been reported that, in diabetes, increased scavenger receptor of modified LDL (CD36) and reductions in the levels of ATP binding cassette (ABC) transporters (ABCA1 and ABCG1), increase macrophage foam cell formation [14]. As shown in Fig 5D, we confirmed both upregulation of CD36, and downregulation of ABCA1 and ABCG1, by both HG and oxLDL. UDCA inhibited the increase in CD36 expression, and enhanced expression of mRNAs encoding ABCA1 and ABCG1, which had been decreased by HG or oxLDL. Similar to ECs, osmotic control of HG, mannitol did not affect the expression of RAGE, inflammatory cytokines, CD36, or ABC transporters in macrophages (Fig 5). These results indicate that UDCA exerts anti-atherosclerotic effects on macrophages via reduction of RAGE expression, inhibition of foam cell formation (by decreasing CD36 and increasing ABCA1 and ABCG1 expression), and downregulation of pro-inflammatory cytokines.

### UDCA reduces atherosclerotic plaque formation in diabetic mice

**Fig 6:**
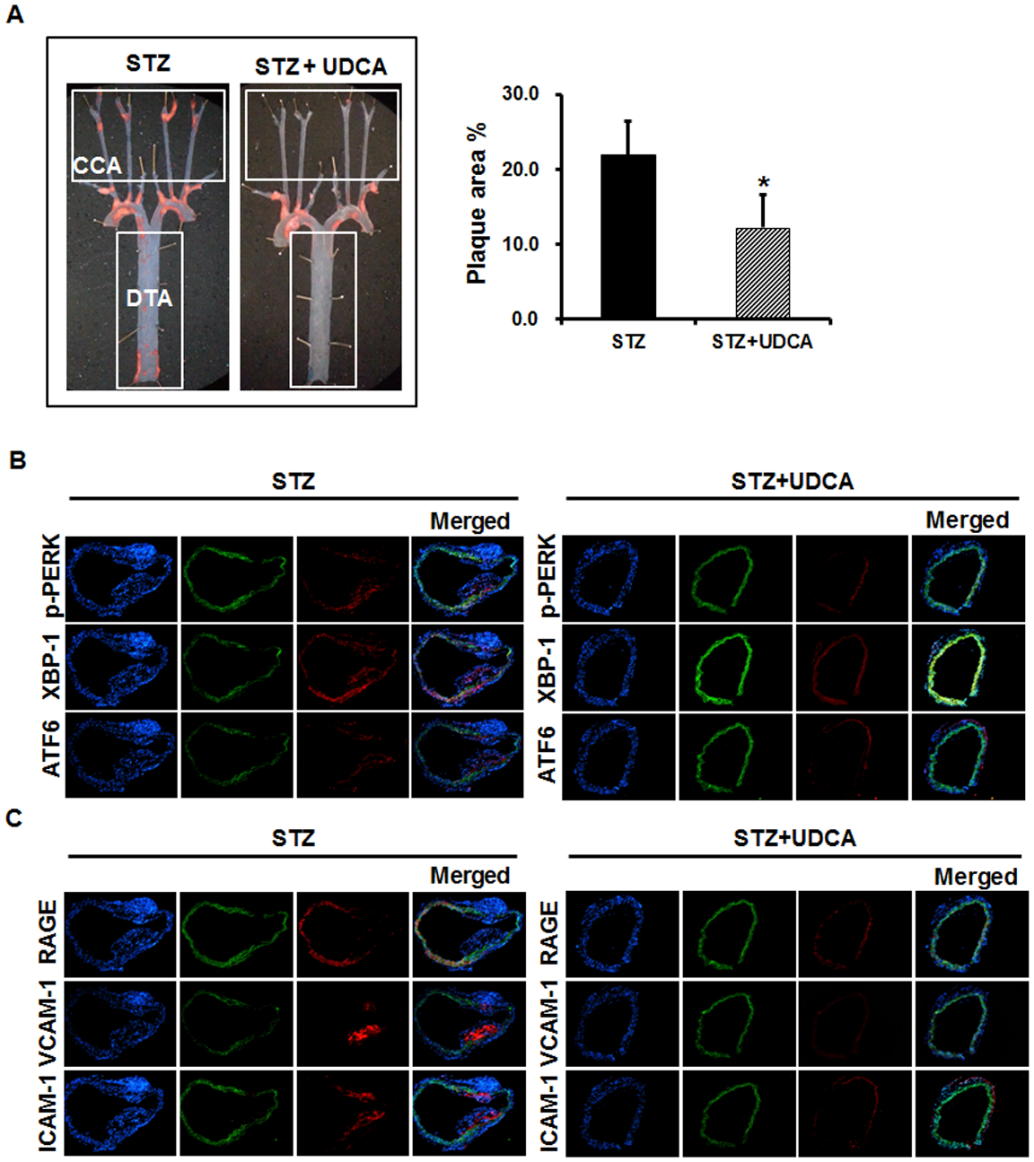
Effects of UDCA on atheromatous plaque formation in diabetic mice. Diabetes was induced in mice via injection of STZ and the animals were fed a chow diet with or without 0.5% (w/v) UDCA. (**A**) Quantification of atherosclerotic lesions. Mouse aortas were isolated and stained with Oil red-O (CA, Carotid artery; DTA, Descending thoracic aorta). Plaque area proportions (% values) in aortas were quantified using the Image J software. * Significant difference at the P < 0.05 based on Mann-Whitney U test (STZ vs. STZ + UDCA). Error bars: SEMs. (**B** and **C**) Immunofluorescence staining of the aorta. ER stress markers (p-PERK, XBP-1, and ATF6) (**B**); adhesion molecules (VCAM-1 and ICAM-1); RAGE; and a macrophage marker (CD68) (**C**), are indicated by red signals. The green color is autofluorescence from elastic tissue in vessel walls. DAPI staining of nuclei is shown in blue; merged images are also presented. Representative images from at least three experiments are shown (magnification, ×100; scale bars, 1 mm).

To explore the role played by UDCA in hyperglycemia-induced atherosclerotic plaque formation, we compared the atheromatous plaque formation of the aorta (via Oil-red-O staining) in STZ diabetic mice and STZ diabetic mice treated with UDCA. In terms of metabolic parameters, although the total cholesterol level tended to be lower in the UDCA-treated diabetic group, no significant difference was evident between the two groups. The HDL levels were significantly lowered in UDCA-treated diabetic group compared to diabetic group. No other parameter differed between the two groups (Table 1). The diabetic group exhibited severe atheromatous plaque formation at the aortic arch and the branches thereof. In contrast, the UDCA-treated diabetic group exhibited significantly less plaque formation, with up to a 40% reduction in plaque size, especially in the carotid artery (CA) and the descending thoracic aorta (DTA) (Fig 6A). To explore whether UDCA could alleviate hyperglycemia-induced ER stress in the aorta of diabetic mice, we measured the expression levels of ER stress markers via immunofluorescence staining. As shown in Fig 6B, the expression levels of p-PERK, XBP1, and ATF6 in the DTA were significantly lower in the UDCA-treated diabetic group than the diabetic group. In addition, RAGE, ICAM1, VCAM1, and macrophage infiltration were detected in the DTA. However, in the UDCA-treated diabetic group, ER stress and the ensuing inflammatory responses were not detectable in non-atherosclerotic areas (Fig 6C). These results suggest that, in diabetic mice, UDCA reduces atherosclerotic plaque formation via inhibition of hyperglycemia-induced ER stress, RAGE expression, and the inflammatory response.

**Table 1:** Metabolic parameters after 10 weeks of study in STZ diabetic ApoE ^-/-^ mice, with or without UDCA treatment (n = 5 per group).

	STZ	STZ + UDCA	P value
Body weight	22.8 ± 0.9 g	22.4± 0.6 g	0.4524
Blood glucose	449 ± 32 mg/dl	395 ± 25 mg/dl	0.2778
Total cholesterol	984 ± 173 mg/dl	676 ± 73 mg/dl	0.2103
HDL	126 ± 20 mg/dl	67 ± 3 mg/dl	0.0045
Triglyceride	95 ± 9 mg/dl	96 ± 17 mg/dl	0.5000

Data are expressed as mean ± SEM. Values were measured in plasma samples from fasting animals. Quantitative variables were compared using the nonparametric Mann-Whitney U test. Differences between groups were considered to be significant at P < 0.05.

## Discussion

**Fig 7:**
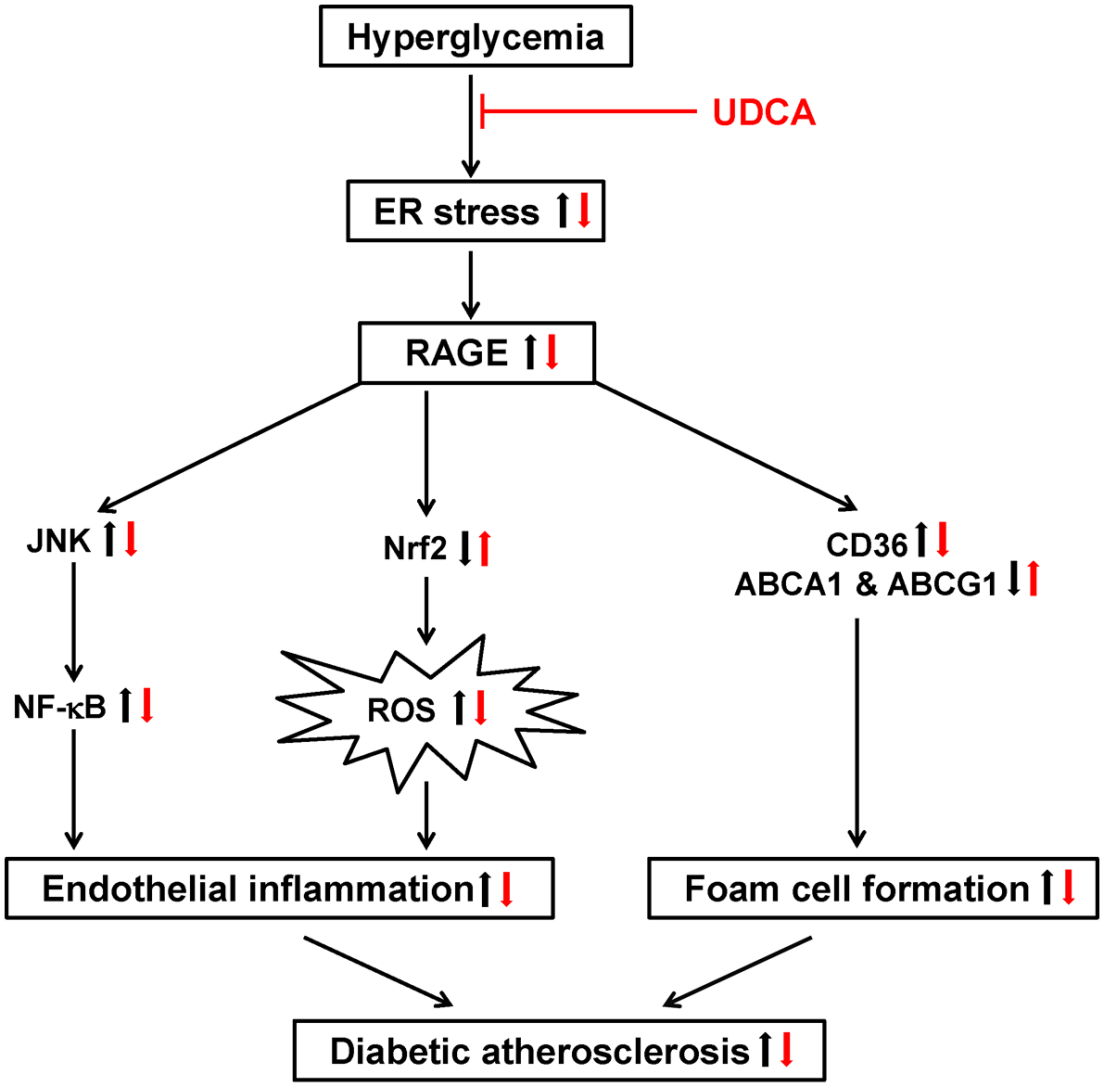
Proposed actions of UDCA in diabetic atherosclerosis. The anti-atherogenic actions of UDCA in diabetic atherosclerosis: In ECs, UDCA inhibits RAGE signaling and inflammatory responses (including NF-κB activation) by blocking hyperglycemia-induced ER stress and inhibiting ROS production by increasing the Nrf2 level. In macrophages, UDCA inhibits foam cell formation via downregulation of CD36 and upregulation of ABCA1 and ABCG1, triggered by inhibition of RAGE signaling. UDCA also reduces the levels of pro-inflammatory cytokines (IL-1β and IL-6) induced by hyperglycemia or oxLDL.

The principal novel finding of the present study is that UDCA exerts anti-atherogenic effects in diabetic atherosclerosis. First, UDCA suppressed the inflammatory response caused by hyperglycemia; UCDA blocked ER stress and the downstream signaling pathway thereof in ECs and ApoE^-/-^ mice. Second, the expression levels of RAGE and the ligand thereof were reduced in UDCA-treated ECs, at least in part via inhibition of JNK and NF-κB signaling. Third, HG-induced ROS production was inhibited in UDCA-treated ECs, via increased Nrf2 expression. Fourth, UDCA reduced foam cell formation via upregulation of ABCA1 and ABCG1 expression, reduction of hyperglycemia-induced RAGE expression, and suppression of macrophage inflammatory responses. Consequentially, compared to STZ-injected diabetic ApoE^-/-^ mice, UDCA-treated diabetic mice experienced significant reductions in atherosclerosis (Fig 7).

Hyperglycemia and dyslipidemia, specific conditions associated with diabetes, have been associated with elevated levels of ER stress [9, 29]. Previous studies have shown that ER stress was increased at all stages of atherosclerosis, and in the arterial endothelium in regions susceptible to atherosclerosis [10, 30]. In our present study, we show that UDCA blocks hyperglycemia-induced ER stress and the downstream inflammatory response thereto in both ECs and aortic tissues of diabetic ApoE^-/-^ mice.

Additionally, we found that RAGE was induced by ER stress and that UDCA inhibited the expression of both RAGE, and the ligands thereof, induced by hyperglycemia or ER stress. In animal studies, RAGE and the ligands thereof were present at higher levels, and accelerated atherosclerotic lesion development, under diabetic conditions [31]. Also, RAGE deletion [28] or blockade [using a soluble AGE receptor (sRAGE)] suppressed the development of atherosclerotic lesions and stabilized established atherosclerosis in diabetic ApoE^-/-^ mice [26, 32]. Very recently, we showed that blocking of RAGE with sRAGE suppressed disturbed flow-induced atherogenesis [24]. In this context, inhibition of RAGE signaling is being targeted to prevent and treat the vascular complications of diabetes. Our findings also suggest that UDCA could serve as a potential anti-inflammatory agent to treat ECs under hyperglycemic conditions.

RAGE-ligand interactions also trigger NF-*κ*B activation and induce the inflammatory response in various cell types. NF-*κ*B is a key transcriptional regulator playing a central role in vascular inflammation. Several studies have shown that ER stress triggers activation of NF-*κ*B and JNK via the IRE1 pathway of UPR [33, 34]. We found that subsequent activation of transcription factor NF-*κ*B and expression of VCAM-1, ICAM-1, and MCP-1 under hyperglycemic conditions were significantly suppressed by UDCA, in turn inhibiting monocyte binding to ECs. Although the effects of HG on MAPK activities (those of p38 MAPK, ERK, and JNK) were variable, the JNK pathway is consistently activated under hyperglycemic conditions [35–37]. In the present study, phosphorylation of p38 MAPK, ERK_1/2_ and JNK, was increased by HG. Interestingly, UDCA blocked JNK activation but not p38 MAPK and ERK_1/2_ activation. In addition, HG-induced RAGE expression was blocked by pretreatment with specific inhibitors of JNK and NF-*κ*B. RAGE triggers a positive feed-forward loop, in which inflammatory stimuli activate NF-*κ*B, which induces RAGE expression, followed by further enhancement of NF-*κ*B activation[38]. Our results imply that UDCA can block the positive feed-forward loop of RAGE signaling in ECs under HG by suppressing ER stress and downstream signaling pathways including JNK and NF-*κ*B activation.

Hyperglycemia can promote intracellular glucose metabolism, triggering overproduction of superoxide anions by the mitochondrial electron transport chain [39]. Oxidative stress is recognized as a key player in the development of diabetic vascular complications [40–43], and hyperglycemia is the principal trigger of the ROS synthesis involved in AGE formation [44] and ER stress [45]. Nrf2 is a key regulator of antioxidant systems under diabetic conditions; many antioxidant enzymes, including HO-1 and thioredoxin reductase [46], are regulated by this protein. In the present work, we show that UDCA significantly reduces ROS production and induces expression of the antioxidant gene HO-1 in ECs under hyperglycemic conditions; this was associated with increased nuclear translocation of Nrf2.

In addition to endothelial dysfunction, macrophage-foam cell formation is an early event in atherosclerosis. Interaction of RAGE with the ligand thereof, S100B, attenuated ABCA1expression in monocytes and accelerated foam cell formation under hyperglycemic conditions [47]. Other studies found that macrophages from Type 2 diabetic db/db mice exhibited decreased ABCG1 expression and increased levels of the scavenger receptor CD36, enhancing foam cell formation [14]. Additionally, RAGE suppresses macrophage cholesterol efflux and probes the mechanisms by which RAGE downregulates ABCA1 and ABCG1 [48]. We obtained similar results, in that hyperglycemia enhanced RAGE and CD36 expression and decreased ABCA1 and ABCG1 expression. However, macrophages treated with UDCA exhibited lower-level RAGE and CD36 expression, whereas the expression levels of ABCA1 and ABCG1 were increased compared to the HG control group. Our results suggest that UDCA reduces foam cell formation via downregulation of CD36 and upregulation of ABCA1 and ABCG1, caused by inhibition of RAGE signaling in macrophages.

Consistent with data of a previous study, we observed increased atheromatous plaque formation in STZ-induced diabetic mice; elevated ER stress marker levels; subsequent expression of RAGE, VCAM-1, and ICAM-1; and inflammatory cell infiltration in aortic tissues. In contrast, in the UDCA-treated diabetic group, atheromatous plaque formation was significantly reduced in the CC arteries and the DTA. ER stress markers and the inflammatory response were either undetectable or significantly reduced in the UDCA-treated diabetic group. In lipid profiles in mice, the HDL levels were lowered in UDCA-treated diabetic group, even though atherosclerotic plaque formation was significantly reduced in UDCA-treated diabetic group compared to diabetic group. These results can afford explanation that anti-atherogenic effects of UDCA on diabetic atherosclerosis are not resulted from changes in cholesterol levels. Therefore, our data suggest that UDCA exerts anti-atherogenic effects, inhibiting the development of early atherosclerosis caused by hyperglycemia, by blocking ER stress, RAGE signaling, and the downstream inflammatory response. As limitations of our study, we could not investigate detailed mechanisms on how ER stress activates RAGE and the functional mechanism of UDCA in lipid metabolism in this study. Further studies are required to elucidate these concerns, which may increase our understanding in anti-atherogenic activities of UDCA.

In conclusion, we found that UDCA exerts anti-atherogenic effects on both ECs and macrophages by blocking RAGE signaling. In ECs, UDCA inhibits endothelial dysfunction by inhibiting ER stress, reducing RAGE expression, inhibiting the inflammatory response (including NF-κB activation), and suppressing ROS production, by increasing Nrf2 levels under hyperglycemic conditions. In macrophages, UDCA inhibits RAGE expression, the expression of pro-inflammatory cytokines, and foam cell formation, by upregulating ABCA1 and ABCG1. This suggests that UDCA, a commonly used chemical chaperone, could serve as a potential therapeutic agent for prevention or treatment of diabetic atherosclerosis.

## Supporting Information

S1 Fig Effects of UDCA on ER stress induced by TG in endothelial cells. (DOCX)

S2 Fig Effects of UDCA on antioxidant gene expression in endothelial cells. (DOCX)

S3 Fig Effects of UDCA on endothelial proliferation and eNOS expression. (DOCX)

S1 Table Primers for RT-PCR. (DOCX)
