# Supplementary material for: Ursodeoxycholic Acid (UDCA) Exerts Anti-Atherogenic Effects by Inhibiting RAGE Signaling in Diabetic Atherosclerosis
Source: PLoS One. 2016 Jan 25;11(1):e0147839. doi: 10.1371/journal.pone.0147839 (PMC4726772; doi:10.1371/journal.pone.0147839)
Supplement: S1 Fig — (DOCX) [file pone.0147839.s001.docx]

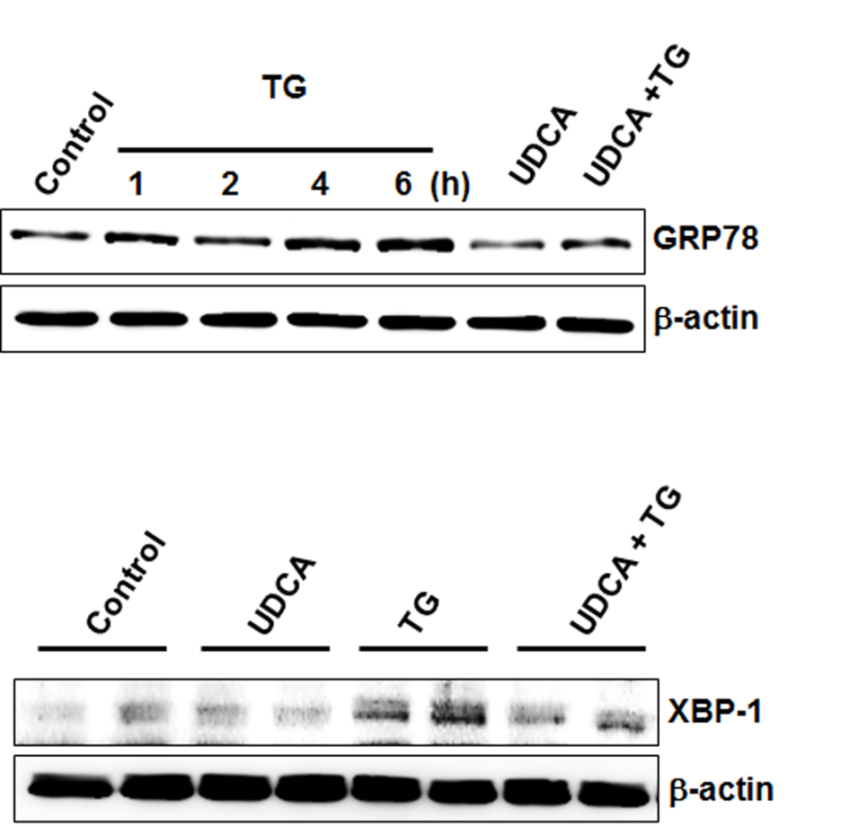


**S1 Fig. Effects of UDCA on ER stress induced by TG in endothelial cells.**

HUVECs were incubated with TG for the indicated times after pretreatment with UDCA. Western blotting was used to detect GRP78 and XBP-1, markers of ER stress. Representative images from at least three experiments are shown.
