# Supplementary material for: Ursodeoxycholic Acid (UDCA) Exerts Anti-Atherogenic Effects by Inhibiting RAGE Signaling in Diabetic Atherosclerosis
Source: PLoS One. 2016 Jan 25;11(1):e0147839. doi: 10.1371/journal.pone.0147839 (PMC4726772; doi:10.1371/journal.pone.0147839)
Supplement: S2 Fig — (DOCX) [file pone.0147839.s002.docx]

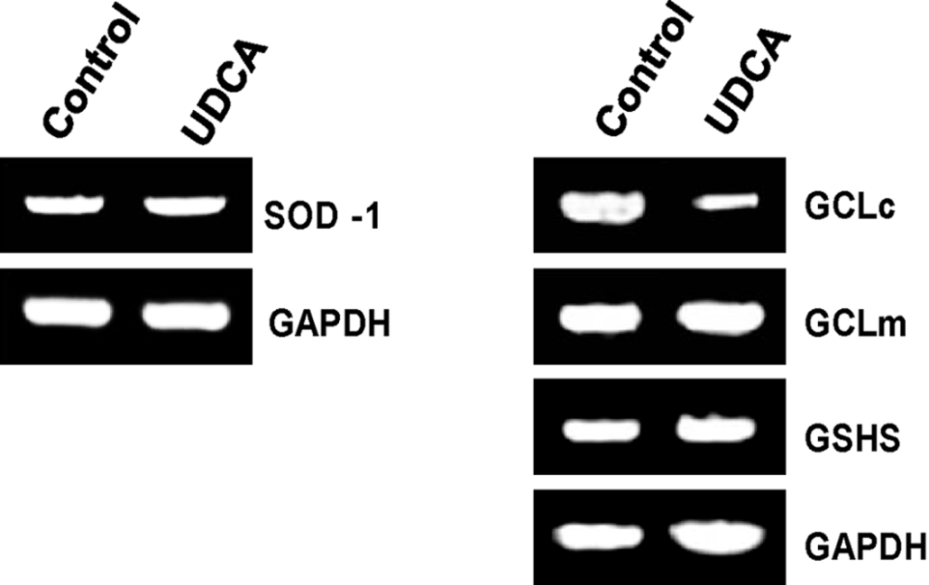


**S2 Fig. Effects of UDCA on antioxidant gene expression in endothelial cells**.

Total RNA was isolated from HUVECs at 8 h after UDCA treatment and the levels of mRNAs encoding anti-oxidant proteins including SOD-1, the catalytic subunit of GCL (GCLc), the modifier subunit of GCL (GCLm), and GSHS, were measured via RT-PCR. Representative images from at least three experiments are shown.
