# Supplementary material for: Ursodeoxycholic Acid (UDCA) Exerts Anti-Atherogenic Effects by Inhibiting RAGE Signaling in Diabetic Atherosclerosis
Source: PLoS One. 2016 Jan 25;11(1):e0147839. doi: 10.1371/journal.pone.0147839 (PMC4726772; doi:10.1371/journal.pone.0147839)
Supplement: S3 Fig — (DOCX) [file pone.0147839.s003.docx]

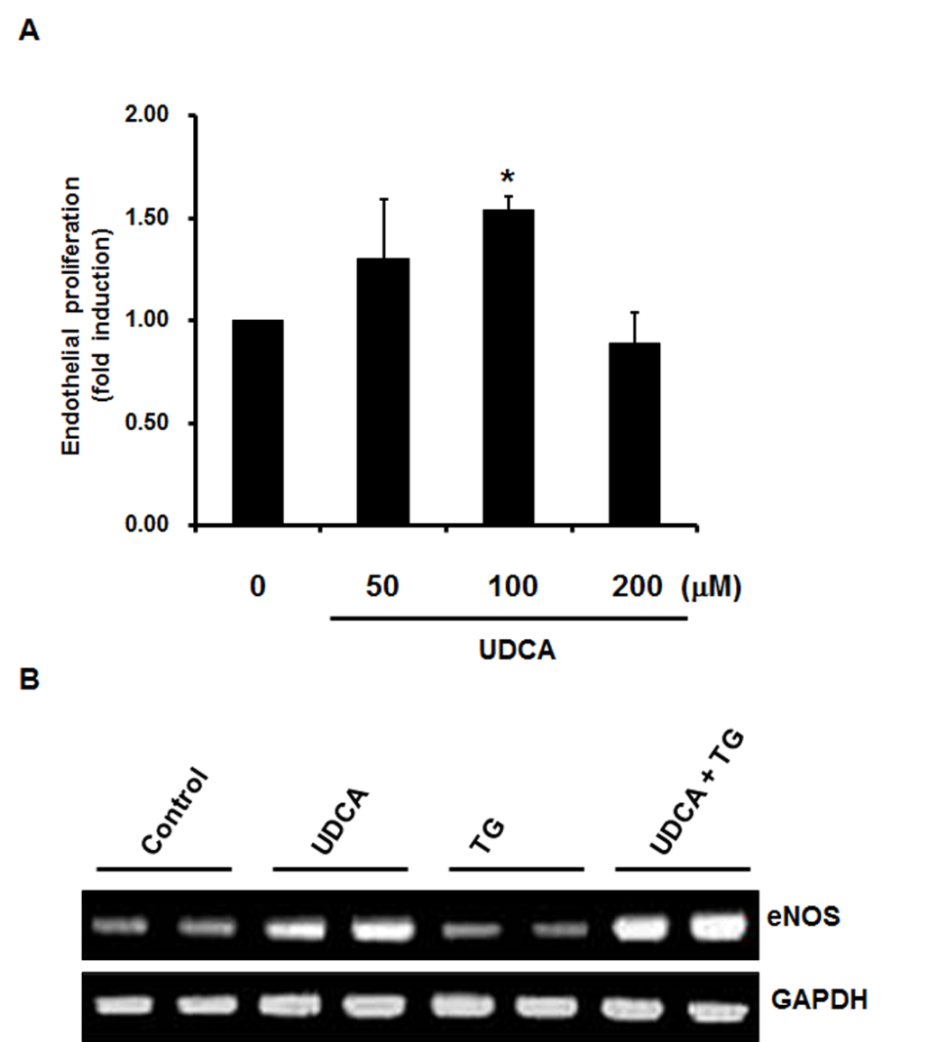


**S3 Fig. Effects of UDCA on endothelial proliferation and eNOS expression.**

(**A**) HUVECs were plated in a 96-well plate at 2.5 × 10^3^ cells/well and treated with various concentrations of UDCA for 24 h. Endothelial proliferation was measured using the MTT assay. * Significant difference at the P < 0.025 based on post-hoc Mann-Whitney U test following a Kruskal-Wallis test (*, Control vs. UDCA). Error bars: SEMs. All experiments were performed at least three times. (**B**) The levels of mRNA encoding eNOS were determined via RT-PCR upon growth with TG for 8 h after UDCA treatment.
