# Supplementary material for: Ursodeoxycholic Acid (UDCA) Exerts Anti-Atherogenic Effects by Inhibiting RAGE Signaling in Diabetic Atherosclerosis
Source: PLoS One. 2016 Jan 25;11(1):e0147839. doi: 10.1371/journal.pone.0147839 (PMC4726772; doi:10.1371/journal.pone.0147839)
Supplement: S1 Table — (DOCX) [file pone.0147839.s004.docx]

**S1 Table. Primers for RT-PCR**

|  | **Primer** | **Sequences** |  |
| --- | --- | --- | --- |
|  | Human RAGE | Sense: AGCGGCTGGAATGGAAACTGAACA |  |
|  |  | Anti-sense: GAAGGGGCAAGGGCACACCATC |  |
|  | Human S100A12 | Sense: TTGAAGAGCATCTGGAGGG |  |
|  |  | Anti-sense: CTACTCTTTGTGGGTGTGG |  |
|  | Human VCAM-1 | Sense: CCGGATTGCTGCTCAGATTGGA |  |
|  |  | Anti-sense: AGCGTGGAATTGGTCCCCTCA |  |
|  | Human ICAM-1 | Sense: GGCCTCAGTCAGTGTGA |  |
|  |  | Anti-sense: AACCCCATTCAGCGTCA |  |
|  | Human MCP-1 | Sense: AGCATGAAAGTCTCTGCCGCCCTTCTG |  |
|  |  | Anti-sense: ATTACTTAAGGCATAATGTTTCACA |  |
|  | Human Nrf2 | Sense: ACACGGTCCACAGCTCATCAT |  |
|  |  | Anti-sense: TTGGCTTCTGGACTTGGAAC |  |
|  | Human HO-1 | Sense: TTCTTCACCTTCCCCAAC |  |
|  |  | Anti-sense: GCATAAAGCCCTACAGCAAC |  |
|  | Human SOD-1 | Sense: GTGGGGAAGCATTAAAGGACTGAC |  |
|  |  | Anti-sense: CAATTACACCACAAGCCAAACGAC |  |
|  | Human GCLc | Sense: CTGTTGCAGGAAGGCATTGA |  |
|  |  | Anti-sense: CGAAATTCTACTCTCCATCCAATGT |  |
|  | Human GCLm | Sense: CAGTTGACATGGCCTGTTCAG |  |
|  |  | Anti-sense: TCAAATCTGGTGGCATCACAC |  |
|  | Human GSHS | Sense: CAGCGTGCCATAGAGAATGA |  |
|  |  | Anti-sense: TTCAGGGCCTGTACCATTTC |  |
|  | Human eNOS | Sense: TGATGCATTGGATCTTTGGA |  |
|  |  | Anti-sense: CCATGTTACTGTGCGTCCAC |  |
|  | Human GAPDH | Sense: GAGTCAACGGATTTGGTCGT |  |
|  |  | Anti-sense: TTGATTTTGGAGGGATCTCG |  |
|  | Mouse IL-1β | Sense: ACTCATTGTGGCTGTGGAGA |  |
|  |  | Anti-sense: TTGTTCATCTCGGAGCCTGT |  |
|  | Mouse IL-6 | Sense: GCCAGAGTCCTTCAGAGAGA |  |
|  |  | Anti-sense: GGTCTTGGTCCTTAGCCACT |  |
|  | Mouse ABCG1 | Sense: GTACCATGACATCGCTGGTG |  |
|  |  | Anti-sense: AGCCGTAGATGGACAGGATG |  |
|  | Mouse ABCA1 | Sense: CGAGAGAGTACTGCGCTACA |  |
|  |  | Anti-sense: TGGTAGATCTGGGTGGAGGA |  |
|  | Mouse GAPDH | Sense: AACTTTGGCATTGTGGAAGG |  |
|  |  | Anti-sense: ACCATTGGGGGTAGGAACA |  |
|  |  |  |  |
